# Sustainable Innovation through Developing Hybrid Agri-Food Supply Chains: A Case in South-Eastern Spain

**DOI:** 10.3390/toxics10120752

**Published:** 2022-12-02

**Authors:** Irene Navarro-del Aguila, Jerónimo de Burgos-Jiménez

**Affiliations:** Department of Economic and Business, University of Almería, 04120 Almería, Spain

**Keywords:** sustainable innovation, hybrid supply chain, short agri-food supply chain, agri-food supply chain, case study

## Abstract

We analyze the incipient development of short supply chains for fresh fruit and vegetables from conventional agri-food chains, leading to the emergence of hybrid chains. We have sought to answer the research question of how and why hybrid food supply chains has been initiated by studying this phenomenon in south-eastern Spain. We analyzed the 20 main Spanish fruit and vegetable exporters, identifying the 9 that have developed short channels. Subsequently, we conducted a case study of the one considered most relevant, identifying the stages of this short chain, analyzing the tasks to be performed in the different phases of the SCS, studying which are common to both and where the differences in the processes arise. The results show the synergies that make them coexist successfully, such as the sharing of personnel, infrastructures and services, complementarity in the product range, thus avoiding food waste, or knowledge of consumer tastes and needs.

## 1. Introduction

Agri-Food Supply Chain (ASC) has undergone many modifications throughout history; it was originally local due to production, information and transport constraints. The advances in plant nutrition, crop management technologies, task mechanization and professionalization allowed the increase in agricultural production and surpluses generation with orientation towards external market. The growth of certain crops in geographic areas with favorable conditions has led to the development of local agglomerations of companies, recognized as agri-food clusters [1].

At the same time, food distribution systems have been concentrated to achieve economies of scale to supply population concentrated in large cities. Thus, improvement of agricultural production and transport systems has shaped the current ASC model characterized by mass production and distribution. The dominant ASC model is an extensive network of interconnected operators comprising specialized farmers, warehouses, handling companies, intermediaries, wholesalers, and retailers that bring the product to the final consumer.

Supply Chain (SC) is understood as a series of operations that ensure that goods are produced and distributed in the right quantities, delivered to the chosen locations in the shortest possible time, with the objective of satisfying consumer needs [2]. The ASC needs to add value to the customer [3]. However, in the dominant mass production model, the distribution of added value within the ASC is not well organized, due to the dominant position exercised by large-scale distribution, which leads to a shortening of margins at source and a distancing from the consumer. Agricultural producers need to gain more space by establishing a competitive base through collaboration and integration between chains.

Short Agri-food Supply Chain (SASC) emerge as a more sustainable option for agricultural products. The SASC is an ASC formed by a limited number of economic actors, committed to cooperation, local economic development and socio-economic relations between producers and consumers in a close geographical area [4].

SASC can change the “industrial modes” of food production and develop supply chains that can “shorten” the industrialized, complex and rationally organized Long Food Supply Chain [3]. This achieves greater interaction between farmers and consumers and changes the relationship in the construction of value in such chains.

SSCs are beginning to arouse interest in the literature, as according to [5] they offer solutions that improve logistical sustainability. However, this author finds the main obstacles to be the lack of convenience and the difficulty of finding a trusted farmer, as well as limitations in variety and limited prices, and concerns about food safety control. These authors consider that the growth of SASCs in Spain will come about by satisfying consumer needs and with well-targeted policies. In reality, all these limitations are already solved with hybrid chains in which an SSC coexists in a long chain. These hybrid chains are spreading in the Spain agri-food sector, although this has not been studied previously from this point of view.

ASCs have traditionally been considered as independent models to the SASC [6]. However, some changes in the environment such as the advances in information and communication technologies, as well as the development of economic, social and environmental sustainability practices, have driven the evolution of these models. We highlight a new type of SASC that is based on synergies with traditional long ASC.

Specifically, we focus on the agri-food cluster of fresh fruit and vegetables in Southeast Spain, where traditional agri-food chains have a structure of association of farmer who performs the functions of handler and marketers [7]. It should be noted that Spain is the main European exporter of vegetables, with a high concentration of agricultural companies in south-eastern Spain, which has facilitated the development of a strong cluster with specialized research and transfer centers. In the last decade, some fresh fruit and vegetables producers’ organization have created parallel short channels through which they also sell their products directly to the end consumer through online or physical shops. Although turnover in this short channel is very low compared to traditional channel, these farmers’ organization companies are developing the strategic capacities necessary to satisfy the needs of the end consumer of fresh fruit and vegetables.

This new marketing channel has a hybrid form. On the one hand, it shortens the normal flow of the traditional channel (the farmers’ organization companies reach the end consumer without intermediaries). On the other hand, it is not a direct channel to the consumer (products are delivered by the farmers’ organization, not by an individual farmer). Individual farmers do not have the productive or organizational capacity to supply the needs of the consumers individually (each farmer is specialized in one crop and at one time of sowing) and must collaborate with other farmers to meet the needs of customers. In addition, this new way of marketing from a hybrid chain solves the problems and challenges recently found by [8] of short supply chains such as the need for logistics infrastructure, the importance of social links, the need to diversify distribution channels, and the product-related constraints of being embedded in a much more powerful structure. These problems have not been solved so far in the literature.

The scope of hybrid chains for these highly perishable products was initially conceived for a local approach but has been extended to the entire national territory with relative success. The process of creating short chains initiated by the innovative farmers´ organization who have developed hybrid chains contributes to establishing direct and close relations between producer and consumer. We found in the literature some precedents in the construction of hybrid chain from the retailer side. The construction of a hybrid fresh produce chain in which innovative retailers adopt hybrid supply chain strategies by leveraging their relationships with other links in the chain to shorten the conventional chain generates efficient advantages in time and product variety [9]. According to what we have been able to find, there are no studies that refer to the development of new ways of managing relations with the consumer from the grouped producer. This is an important advancement in the SASC, being a relevant topic whose gap we intend to fill.

The main objective of this research is to analyze the process of the emergence of short supply chains within the long agri-food supply chains in the fruit and vegetable cluster of south-eastern Spain. This will help us to find out why and how these chains have started the process of moving closer to the market. An intermediate objective is to analyze the main Spanish fruit and vegetable exporters by identifying those that have developed a short channel. We started from the hypothesis that hybrid supply chains share stages of the chain by analyzing the tasks that have to be performed in the different stages of the SCS, studying which are common to both and where the differences and synergies in the processes with conventional chains arise.

This study represents an evolution in the concept of hybrid chains towards sustainability. There is a new direct chain from a handling-marketing company to consumer. This is a shortening of the traditional chain by eliminating the figure of the retailer and performing the function of supplying the end consumer directly by the SASC, and this has not been studied before from this perspective. We will first analyze the causes that lead to the emergence of hybrid chains in which conventional supply chains are developing a short direct sales channel due to the facilities provided by digitalization and online commerce, as well as the need to create value by increasing commercial margins and providing a solution to the distance between the consumer and the producer. To this end, we will analyze, in depth, the operating structure of an innovative SASC, discovering the synergies between the two coexisting chains that give rise to hybridization. We will review the growth of this paradigm in the sector within the specific agri-food cluster of fruit and vegetables in which it is located, exploring whether this trend can be replicated in the rest of the agri-food industry and thus be extrapolated to other agri-food clusters.

## 2. Materials and Methods

### 2.1. Theoretical Background

Most food sales and distribution are conducted by large conventional food supply chains, which represent a network of food-related organizations within which products move from producers to final customers [10]. These SC are the most cost-efficient for handling large volumes, but they disconnect producers from consumers. In addition, they can also cause problems such as food waste, food safety, environmental damage, unfair distribution of added value and profits among the members of the chain [11]. This situation, together with the growing interest in food quality and local production, increase the importance in SASC. Additionally, social media is an important element to develop and improve relationships with customers [12].

The advancing digitization of ASCs allows SC members to be highly connected, win efficiency and traceability, and respond to customer needs and legal and regulatory requirements [13]. It enables producers to create a channel to communicate and operate directly with consumers. Therefore, there are new market opportunities based on providing accurate information to selected members of the supply chain and consumers. This avoids the communication bottlenecks that can result from an information network dominated by intermediaries.

Today’s food system faces major challenges in terms of sustainable development in economic, social, and environmental dimensions [14]. These challenges are often associated with the dominant long ASC with industrialized production processes and longer and less transparent supply chains. For example, on the social and economic side, SASC have the potential to improve farm incomes, promote sustainable farming systems, and contribute to local economic development [15,16]. On the environmental side, SASC may decrease food waste, reducing the time from when the farmer picks the product until it reaches the consumer. Time reduction requires the coordination of the different actors involved in the SC and this is easier in SASC where there is more proximity and less intermediaries [17]. Time reduction will be especially useful in perishable product chains that have to make a great effort to maintain the quality of the goods from the beginning of the chain until they reach the point of sale.

A differentiating characteristic of Fruit and Vegetable ASC is that their products are highly perishable. Regarding these types of products, consumers give priority to fresh products with a reasonable price, which implies an accurate supply chain management that becomes even more complex if there are intermediaries involved [18]. The existence of a short life-time implies significant time reductions in storage and transport processes, which can lead to unavoidable losses along the SC.

Consumers are increasingly demanding an orientation towards territorialism in agriculture [19] because they look for freshness, organoleptic properties, contribution to health and support for the farmer in short chains. In this SASC, it is usual to pay attention to seasonality, local and regionalized conditions (for example to manage nutrient cycles), and certifications of origin. Improved information management and the need to increase the value of production for the farmer drives SASC. Their product differentiation relies on spatially linked “consumer-producer relationships” as a basis for trust. The SASC builds value by facilitating direct contact between farmers and consumers, based on trust and honesty and it is characterized by the absence of intermediaries [20].

There is no shared and unique definition of SASC, but all of them highlight the closeness between producers and customers. The length of the SC is determined by the proximity between farmers and consumers and can be assessed through different parameters: (1) by its area of location (geographic); (2) the number of intermediaries needed to access the consumer (steps); (3) the period needed to complete the delivery from farmer to consumer (time); and (4) the type of relations between the members of the SC (communication).

Usually, most of these parameters evolve in a similar way. More geographic proximity is often associated with fewer intermediaries, shorter time to market, and better communication. Thus, each definition usually focusses on one or more of the above parameters. For example, Regulation (EU) No 1305/2013 of the European Parliament And Of The Council defined SASC as: “a supply chain involving a limited number of economic operators committed to cooperation, local economic development and close geographical and social relations between producers, processors and consumers” [21].

Thus, SASCs can be classified according to different criteria such as the spatial proximity of their members [4] distinguishing between:

1. Face-to-face: where producers sell their products directly to the consumer in a face-to-face manner, such as farmers’ markets, farm gate sales, produce collection systems, and box systems.
2. Spatially proximate: where products are produced and sold through local market channels in the specific region of production, including farm shop groups, food outlets, local food retailers, and consumer cooperatives.
3. Spatially extended: where products are sold not only to local consumers but also to consumers in other regions. In this case, labeling and certification schemes could be used to differentiate these products by emphasizing quality such as designation of origin.

If we incorporate other criteria such as the organizational forms adopted and social interactions for performing activities by producers or consumers [22], they differentiate other basic forms of SASCs:

1. Direct on-farm sales: allows face-to-face relations between producer and consumer.
2. Pick-up of own produce in the field: consumers collect produce on their own directly from the field.
3. Box scheme: farmers’ cooperatives and local consumer groups ensure regular procurement of seasonal food sustainably grown in or around the local community.
4. Farmers’ markets: these are markets, usually located in urban areas and held periodically, where a group of farmers come together and where each producer sells his or her agri-food product directly to individual customers.
5. Collective farm shops: farmers come together to jointly set up and run a shop in a market town or in suburban or urban areas where they sell their local produce.
6. Community Supported Agriculture: involves consumers and local farmers participating in a common agreement; consumers commit to buying seasonal food from producers, who deliver it regularly to consumers’ homes.
7. Collective buying groups: organized consumers who choose to buy commonly directly from selected producers.

These SASCs are often presented as an alternative model to traditional SCs that can also coexist with traditional ASCs [3]. However, SCs change in size, shape and configuration, and in how they are coordinated, controlled, and managed in order to adapt to the market [23]. Farmers or farmers’ associations can sell a part of their products to wholesalers (traditional channel) and sell another part directly to consumers through physical or online shops. In addition, producers can also directly serve to other customers such as hotels, restaurants, and catering firms (https://www.canalhoreca.com, accessed on 21 October 2022). This results in hybrid supply chains [9].

In this sense, ref. [3] present different scenarios for the coexistence of ASC and SASC, depending on the convergence or divergence of interests among them and the lower or higher need to add value. For convergence of interests, they proposed co-operative coexistence strategies such as sharing relations of inputs and processes. For coordinative coexistence, broader strategies are envisaged which include sharing practices, relations, knowledge, values, and processes in order to scale up SASCs or shorten ASCs.

### 2.2. Research Methodology

#### 2.2.1. Context of the Study

Our analysis focused on the Fresh Fruit and Vegetables sector, which in Spain accounted for 8.6% of the total agri-food industry in 2019 [24]. The Spanish Vegetable Supply Chain in recent years (mainly South East Spain) has become the main horticultural supplier in Europe with a 40% share of fresh vegetables [18]. The south-east region of Spain has the highest concentration of greenhouses in the world [25]. Its productivity is 30 times higher than the European average. The success of the Almeria agricultural model is based on the use of simple but highly efficient technologies, such as plastic greenhouses or sandy soil crops. In the Almeria production model, fruit and vegetable crops are grown on small family farms of an average size of 2.5 ha. Production is specialized in eight main crops (peppers, tomatoes, courgettes, cucumbers, aubergines, green beans, watermelons, and melons). Vegetable production takes place mainly during the winter months. Each farmer decides on the variety he wants to sow, choosing one or at most two crops during the year.

In addition, an extensive network has developed in parallel, consisting of handling-marketing companies, wholesalers, transport companies, support organizations (including farmers’ associations, irrigation communities, credit cooperatives, etc.), and different auxiliary industries with specialized research and transfer centers that give rise to an important business cluster [7].

Marketing is conducted through handling-marketing companies with whom the farmers normally maintain stable relationships. These handling-marketing companies receive fruits and vegetables from multiple farmers and prepares them (residue analysis, washing, calibration, packaging, etc.) for delivery to their customers.

These handling-marketing companies play a central role in the agri-food chains of south-eastern Spain as they maintain stable relationships with farmers. Handling-marketing companies are cooperatives whose members are farmers or they are trading companies with close relationships with farmers. In both cases, farmer collaboration relationships cover areas such as the sale of seeds, plant nutrition products, pest treatment, technical advice, price information, etc. Approximately 80% of fruit and vegetable production is exported, a situation that has remained stable over the last 3 years [26].

#### 2.2.2. Methods

In a first stage, we conducted a systematic search for Fruit and Vegetable ASCs that have developed similar SASC in the selected study area. We have tried to test the hypothesis that a trend is beginning in conventional supply chains to develop a short chain that co-exists with the traditional supply chain. This gives rise to a change of paradigm that we call hybrid supply chains.

In order to select the sample, we based it on the 2020 Alimarket ranking of Spanish Fruit and Vegetables exporters and we reviewed specific information of the 20 main vegetable exporters operating in the area. For this purpose, we analyzed the websites of these companies (as all of them have presence on Internet), specific consumer blogs and news in the media. Additionally, we phoned those exporters for which we found no evidence of sell their products directly to end consumers (via digital platform or physical shops).

We identified 9 SASC that have basically emerged in the last 5 years. A detailed study was made for 10 months reviewing online sales platforms of each of them, reviewing their product catalogue, ordering system, delivery logistics, and frequency. Where necessary, a more detailed analysis was conducted with company visits and interviews. Interviewees were carefully selected to ensure that they were people with the capacity to provide comprehensive information. Data has been triangulated where possible with reports in the specialized press and interviews with experts and business associations in the sector. The results obtained have been anonymized to protect the data and the confidentiality of the companies studied.

In a second stage, one of the 9 SASCs identified previously was selected to conduct a case study of a handling-marketing company where a SASC has been developed within a long ASC. This company was selected because it is a reference in the fruit and vegetable sector in the south-east of Spain, characterized by its innovative orientation. Top management was contacted by the middle of 2021 and agreed to participate in the study. The company in question, which is a second-tier cooperative, is interested in studying the improvement of its supply chain in the short term and is even participating in some research projects on the subject.

The case study followed the methodological approach proposed by [27] to analyze innovation in agri-food supply chains: (1) mapping of the current agri-food chain and (2) identification of improvement projects. This methodology is based on the assumption that in an effective value chain, companies do not operate in isolation. Partnerships between companies lead to co-innovation. This is possible when there is a common purpose between stakeholders, compatible structures and processes, opportunities for mutual benefits and cooperation, in an atmosphere of trust and commitment. It is very important that stakeholders have a learning attitude in everything they do together to create a co-innovation environment.

Firstly, we conducted a mapping of the state of the value chain, information flows, and relationships within and between the companies that constitute the value chain analyzing from the input and primary production and the rest of the phases in depth. In order to identify the phases of the SASC, semi-structured interviews and working meetings with the different SASC supply chain professionals in the fruit and vegetable sector were held over a period of 8 months (in a 6-step process).

The key agent study case analysis began at the end of 2021, holding a joint meeting with the company’s middle management and heads of functional areas (business development, operations, quality, logistics, information technologies, marketing and customer service and heads of handling centers in which, the objective of the collaboration was exposed. Subsequently, telephone interviews were conducted with some external suppliers (packaging, seeds, and phytosanitary products), external logistics services provided by the company, in which their collaboration with other agents in the value chain was contrasted. This first step ended with an interview with the business development manager where we reviewed all the information provided by the key agents of the case study and we elaborated the detailed proposal of VC phases.

Later, we conducted semi-structured interviews with competitor companies, selected from the initial sample of the main vegetable exporters operating in the area that had developed a short chain, analyzing their structure and considering the common information provided until an agreed phase proposal was reached.

At the beginning of 2022, we contacted the main association of producers and marketers to consult their opinion according to their experience and knowledge of the sector, generating a new proposal of phases.

Once the above processes had been completed, a group of 3 university professors/researchers who are experts in the sector were contacted and invited to contribute their joint proposal for the links in the fruit and vegetable supply chain.

Subsequently, a global meeting was held via videoconference with representatives of each of the individual proposals to reach a consensus proposal. The reason for the meeting was explained to them and they were provided with the 4 proposals above. This was followed by a constructive discussion until we came up with a consensual phasing scheme representing the simplified functioning of the short and long chains.

Finally, the research team (university professors who are experts in the sector in coordination with the company’s functional managers) adapted this proposal for terminological adequacy and for generalization to other similar organizations.

## 3. Results

### 3.1. Hybrid Agri-Food Supply Chains Identified in South-Eastern Spain

Below we present the results of the study of the Fruit and Vegetable Supply Chains included in the list of the 20 main exporters located in the study area that have developed SASC in the last 5 years, becoming hybrid chains. Their main characteristics have been analyzed and are shown in Table 1, which summarizes the hybrid agri-food chains in south-east Spain. These characteristics are the following: type of matrix chain, type of short chain, year of implementation of the short chain, range of products offered, whether it allows changes in the composition of shipments, frequency of shipments, whether it allows collection at origin without having to contract transport, whether it has the cost of transport included, the level of prices, whether it conducts promotions, the quantity shipped, the shipping area, whether it has online sales, whether it has a website or social networks, and whether it offers the possibility of subscription. A high level of digitalization with online sales can be seen in most cases. The majority use of the box scheme, similarity in the frequency of deliveries, and other characteristics will be explained in detail below. The companies have been anonymized to maintain their confidentiality.

**X1:** Second-degree cooperative, with the association of numerous first-degree cooperatives. It launched its online sales in 2019, according to news from the specialized magazine [28]. Through its website, customers located in mainland Spain can purchase a range of fruit and vegetable boxes containing 5/6 kg. of produce from one or more of the member cooperatives. The box can be single-product (with one or several varieties), multi-product with a fixed or variable composition, with organic and/or conventional produce. The price is affordable and stable. Specific products have their own profile on most social networks. The box is made of compact cardboard and the most vulnerable products are packed in paper or compostable plastic bags. It makes a competitive offer with adjusted prices, in which transport is always included. Delivery takes place on Thursdays, and orders are accepted until the previous Sunday. They have a system of order subscriptions that allows them to build customer loyalty. They do not operate in summer months.

The variable multi-product box offered by this cooperative requires special attention because of its originality and the possibilities of matching fruit and vegetables supply and demand. One of the criteria for selecting the composition of the variable multi-product box is the overproduction of a specific product or size at certain times of the season. Initially, they conducted a test phase in which they were only marketed to the cooperative’s employees; it was a success and they created an electronic platform for weekly marketing with direct delivery to consumers’ homes.

**X2:** Cooperative specialized in marketing of fresh tomatoes. It was one of the first to launch its online sales in 2018, according to Origen, the magazine of rural flavor [29]. It offers vegetable boxes of different weights (from 3 to 6 kg.) where tomatoes of Premium varieties (such as RAF, Adora, Rebelión, Rosa Tradición...) play a predominant role. It also offers boxes of vegetable combinations and tomato mixes, for some products with organic certification. It allows a certain flexibility in choosing the quantity of product to buy, and ordering time (allowing it to reach the consumer in the shortest possible time after harvesting). Delivery is available two days a week, Tuesdays and Thursdays. The price of delivery is not included in the sale price; it is calculated according to destination, which can only be national or picked up by the customer at the company’s own facilities. It does not operate in the summer months.

**X3:** Business group dedicated to the commercialization of fruit and vegetables in more than 30 countries. Its online sales started in 2011 with reduced activity and it has renewed in 2021 [30]. Pioneer in selling RAF tomatoes and other products through its website. They have expanded their catalogue of products and formats. In addition to Raf tomato, with or without extra virgin olive oil from Almeria, and multi-product boxes of vegetables, they have introduced mixed boxes of tomato and tropical fruits such as avocado, mango, or Kumquat. Their logistics are flexible as you can choose your destination or pick up from one of the group’s logistics centers. The cost of transport is not included in the price either, it is calculated according to the destination, which has to be national. It does not operate in the summer months.

**X4:** Value chain specialized in organic farming. It has an online shop where consumers can buy fruit and vegetables of pre-prepared and pre-cooked convenience food products (grated tomato, gazpacho) since May 2018 according to news in the specialized press. Customers can buy products available in the digital platform with flexibility (from one package to a complete basket). It can be picked up at the warehouse (free of charge) or shipped throughout the Iberian Peninsula, including shipping costs if the minimum purchase amount is exceeded (for example 25€). The order is delivered by a courier company that sends the product to the customer’s address within 24 h.

**X5:** Important cooperative that started its online sales in 2020, according to news communicated by Cadena Ser [31]. It has an online shop where you can buy its fresh vegetables and fruits packed in its own branded boxes with an assortment of standardized product combinations, or you can buy any weight of products from the cooperative’s catalogue of independent products offered to combine them. The frequency of deliveries is daily, with a commitment to next day delivery. They allow collection from their locations or home delivery throughout the peninsula, free of charge above a certain amount. It operates all year round, although there are fresh vegetables whose availability is limited to the production season.

**X6:** First degree cooperative with almost 1000 farmer members, characterized by bringing the farmer closer to the market. In recent years, it has strengthened its offer of organic and integrated production (more respect for the natural environment, but without certification). It has opened physical shops (supermarket type) in two of its four locations, the last one in April 2019, and is preparing a future online sale of its own products. In the physical shops, the general public can buy the company’s horticultural products, including pre-prepared and canned products, as well as a limited range of external products (as a supermarket). It is open all year round, although during the summer season (when production is lower, opening hours are reduced).

**X7:** Limited company marketing and exporting melons, brassica, and lettuce. It emphasizes differentiation through traditional varieties where flavor is enhanced. It has recently started its online shop with a differentiated range of products for the summer season, mainly melons in multiple formats, with boxes of one or several varieties weighing between 5 and 10 kg. Home delivery in mainland Spain with postage included in the sales price. In the winter season it incorporates leafy vegetables and reduces the variety of melons on offer.

**X8:** Fruit and vegetable cooperative with more than 400 farmer members and marketing in more than 30 countries. It currently has four fruit and vegetable shops open for sale to local consumers, the first of which was opened in 2011, in places close to its handling centers where it sells vegetables, fruit and vegetables, conventional and organic, mostly from its farmers as well as its pre-prepared convenience food products (manufactured since 2008 and expanded in 2020), complemented by a reduced range of food products. It has conventional business hours during which orders placed by telephone can be picked up in the shop. It also offers a personalized service for local hotel and restaurants that includes products delivery.

**X9:** Agricultural supply and production business group that is starting to market a small range of pre-prepared convenience food products (canned vegetables) online on a specific platform and is planning to include some fresh vegetables.

Of the 20 main vegetable exporters in Spain, 45% have already developed a SASC, thus initiating their contact to the end consumer, confirming the hypothesis of what is beginning to be a significant trend among traditional ASC in the area. Furthermore, the rest of the main Spanish vegetable exporters that have not yet started, are planning to do so in the near future.

### 3.2. Case Study: Analysis of the Different Stages of the Chain and Its Innovations

We selected X1 for the case study for the reasons already given. Table 2 shows the supply chain phases proposed by each of the four groups of supply chain professionals who participated (four columns on the left): key agents in the case study, competitor companies, marketers’ association, and expert university professors, followed by the proposal elaborated in the global meeting in which representatives of each of the above proposals were convened to reach a consensus proposal. Finally, we drew up our proposal with the support of university professors who are experts in the sector in coordination with the company’s functional managers. The final proposal attempts to use appropriate terminology that can be widely interpreted and used by organizations in the sector.

Next, we describe each phase of the ASC in our case study, paying special attention to the tasks that need to be conducted in each phase, reviewing which are common to traditional and SASC, and in particular, the adaptations that need to be made in some processes that are susceptible to generating incremental innovations:

**Input:** This is the reception of inputs from the agricultural production activity and packaging materials for marketing. Intensive fruit and vegetable production requires a wide range of goods and services such as irrigation systems, agricultural machinery, plastics and packaging, seeds, seedlings, insects (for integrated pest management), plant nutrition products, phytosanitary fertilizers and technical assistance. In addition, materials for marketing preparation, cardboard, and plastic packaging are included.

The inputs for agricultural production are usually common to both chains, but the packaging for processing and transport in the two chains is often different, due to differences in proximity, transport temperature, and product mix in the short chain.

**Primary production (agricultural):** consists of the production process of the horticultural crop (normally in greenhouses). It requires the following tasks: farm preparation, planting, crop growth, harvesting and end of crop [25].

The agricultural production model used is based on the combination of simple but very efficient technologies, such as drip irrigation, plastic greenhouses, sand-soil technique, biological pest control, which requires continuous training, multiple inputs and technical advice. This phase of agricultural production is the same for both types of ASCs (traditional and short).

**Transport of products to the handling centers and processing:** In this phase, which is common to both ASC the operations of coding, sorting and storage of farmer’s fruit and vegetables is conducted. Handling centers are typically close to farms and each farmer brings its harvest with his own vehicles to its usual handling center. In the conventional channel, the handling and packaging centers usually perform the following processes: reception of the fruits and vegetables from the greenhouses, storage and refrigeration, cleaning, classification, packaging, palletization, storage and refrigeration, and transport to their destination [25]. In both ASCs, a rigorous traceability process is conducted that follows the origin and destination of all the fruits and vegetables that enters and leaves the SC by a code assigned at the reception of each farmer’s harvest.

**Order preparation:** This phase is different for SASC and conventional ASC. In SASC, packaging and outbound logistics requires centralization in a dispatch center. Therefore, sometimes selected fruits and vegetables must be transported from the center of origin to the common dispatch center. This is followed by packaging, palletization, storage, and cooling. However, in the conventional ASC, internal transport is usually not necessary. Packing, palletization, storage, and refrigeration are conducted directly in the same center where the farmer takes his harvest. In ASCC, packaging is usually standardized, but when the product is a box with a mixture of fruits and vegetables, its content often varies according to crops’ seasonality.

In long ASC, order preparation is standardized and more easily planned, although requires different packaging for each product and customer. The order preparation process is highly automated and involves strict refrigeration control to keep the cold chain of the food.

**Digital marketing and Information Technologies (IT) service team:** This phase is transversal to the entire ASC. In traditional ASC, digital marketing is mainly informative. Commercial activity is focused on high-volume customers, with personalized relationships planned for the medium and long term, with frequent framework agreements for supplying customers/companies throughout the year and in their own packaging. IT allows different levels of integration with customer depending on the trust and stability of their relationships.

In SASCs, there is direct interaction with consumers, generating a large number of relationships and a greater flow of information. They plan and execute promotional campaigns and communication strategies in social media such as Facebook, Instagram, updated blogs, newsletters, and videos, using influencers to help sales. They are in charge of informing about new products by defining strategies, objectives, and priorities and communication managed into a marketing platform. They also have powerful tools for market analysis. Planning needs have to be reviewed and updated at very short notice (weekly) to incorporate information on supply (agricultural production and long-channel excess) and demand (orders, preferences, complaints and other end-consumer feedback).

**Distribution:** this essentially consists of bringing the product from the handling center to the customer. Delivery to customers is usually subcontracted to specialized transport companies, although it follows different processes depending on the type of short or long chain. More than 80% of traditional ASC is exported, requiring long-distance transport, normally with road transport companies with large fleets of refrigerated trucks, generally with products of the same category. However, delivery to customers in SASC is conducted by small delivery vans (normally not refrigerated) contracted to external transport agencies, as these are national deliveries (if consumers are employees, internal transport is usually used).

**Consumers and customer service:** This basically consists of attending to the needs of customers. In this stage there are major differences since in the long ASC, the main customers are large distribution chains and wholesalers. The communication system is relatively formal [18]. In contrast, in SASC, the relationship is direct with many consumers. The communication is managed though a digital platform that makes it easier to maintain a bidirectional and agile communication with the consumer and to build stable relationships with them.

## 4. Discussion and Limitations

In the south-east of Spain, some of the main producer groups or marketing companies of fruit and vegetable products are developing short agri-food channels for fresh fruits and vegetables (pre-prepared convenience food products of their own production) as a viable initiative for the domestic market. The national (local) customer has come to value freshness and flavor with guarantees of food safety and compliance with all the quality protocols required by the international market.

The first advances were made through physical shops, but since 2018, handling-marketing companies (with stable supply and cooperative relationships with producers of fresh fruit and vegetables) are betting on virtual shop to develop SASC. In this regard, ref. [4] suggested that the widespread use of information and communication technology and especially social media can open up opportunities for ASCs to build SASCs. The model of supply chains based on E-Commerce has also been studied by [32] who highlights that most studies in this field have been conducted in China, the United States, and the United Kingdom. Although this model is rarely studied in the literature, it is of great importance due to the accessibility it allows from the customer to the company, causing a great effect on business behavior. Specifically, he states that the fresh food trade has become heavily involved in e-commerce, eliminating distances, intermediaries, and the integration of the old supply chain. Even though fruits and vegetables are one of the most important products in the Spanish agri-food sector, due to their perishable nature, they are among the food products that are least sold online.

Building trusting relationships in an online environment is a key element for the adoption and development of e-commerce [33]. The inability to experience the product and judge quality prior to purchase is also one of the main barriers to the growth of e-commerce. Large handling-marketing companies have a certain prestige in the agri-food marketplace with quality certifications of their processes (demanded by foreign markets) and proven traceability systems that confer security and confidence. This safety perception gives these new SASCs a certain competitive advantage over other short chains that sell fruits and vegetables online without being integrated in a traditional ASC.

**Figure 1:**
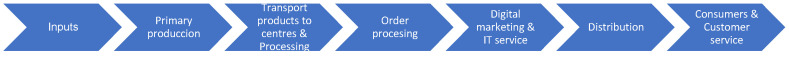
Stages identified in SASC.

After a process of proposal and discussion with various types of experts, we have identified seven stages in this new SASCs that share part of the activities with the conventional ASC. It is remarkable that, depending on the composition of the experts involved, the proposals broke down in greater detail the stages in which these experts were involved. For example, in the first proposal where the seed companies participated they proposed differentiating the stage of seed selection from the rest of the supplies. The final proposal is the one shown in the right-hand column of Table 2 and presented sequentially in Figure 1.

The first three stages of the process (procurement of inputs, primary production, and transport to the handling center) are essentially common to both channels. However, the packaging used for the short channel is usually different from that used for the long channel. The divergences between the two channels start to occur at the order processing stage.

From order processing, the functioning of both channels is different because the competitive orientation of the products changes. The long channel is in a phase of maturity of the product life cycle where a large part of the products in the long channel are considered commodities. Cost efficiency is pursued and a high volume of production is worked with [34]. However, in SASCs, the products, in many cases, are not standardized and allow frequent changes. Therefore, more emphasis is placed on differentiation (by freshness and quality of the product).

Although the storage of handled products is performed in shared facilities between the short and long channels, physical distribution is outsourced and performed by specialized companies. Finally, digital marketing and customer service efforts support both channels, although digital marketing is more focused on SASC. In the long ASC, the interaction of the sales team (by telephone and in person) is more common.

In the case study, we have identified that there are synergies between the short and the long channel. In addition to trust that comes from size and certification of the handling-marketing company, the most important synergies are based in co-operative coexistence strategies [3]: resources are shared in tasks such as planning and monitoring of farmers’ crops, reception and storage of goods, machinery and handling personnel, as well as traceability and quality control systems. There are also synergies in corporate communication on the sustainability of production, product goodness, food safety and hygiene, and respect for the environment. The SASC offer important opportunities for more advanced coordinative existence strategies [3]: short channel can complement the long channel by providing information on consumer tastes and needs [35]. Although SASC does not seem to be a good alternative for ugly food, it can increase the value of some high-quality products that do not fit the wholesaler’s specifications (in terms of size, variety, color) but may even have superior characteristics (e.g., organic origin or enhanced flavor). In this line, especially interesting is the multiple product variable box in order to match what is offered to the market with farmer’s production.

Some of the main innovative fruit and vegetable handling-marketing companies are betting on the short channel in a business venture with strengths and weaknesses. On the demand side, individual consumers have demonstrated their willingness to pay more for high-quality food products [26] and the characteristics of freshness and taste are particularly appreciated by consumers for choosing these perishable products, even over price [36]. In addition, organic production certification is also positively valued by consumers [35] and reinforces the contribution of short channels to sustainability. However, consumers find barriers in the price of transport when it is not included in the price and, in some cases, in a reduced variety of products on offer (which does not meet part of their needs).

On the supply side, the handling-marketing companies that have started the SASC focus on supplying their products to the local/national market. Currently, their priority objective is to meet the needs and tastes of the consumer rather than profitability. These short chains can be extended into medium chains by extending these relationships to regional supermarkets, restaurants, public, and private institutional buyers [36]. This is happening with some companies in the south-east of Spain that have developed their distribution for hotels and restaurants with local companies. Therefore, they are developing hybrid supply chains [3]. However, barriers have been detected, such as the seasonality of production, the reduced range of products, the variable prices of the products on offer, and the difficulty of organizing uniform and quality transport with high variability in the destinations and quantities to be transported. Some of these barriers such as seasonality or lack of variety have been identified as barriers to customer retention in other short chains in other environments such as California [37]. In order to reach the consumer, handling-marketing companies can collaborate with specialized external companies (e.g., product design with seed manufacturers or digital marketing projects). It is highlighted that in the distribution phase up to the consumer, no company has opted for the development of its own transport network up to the customer and this management is completely outsourced to specialized companies (for the companies that offer it).

The growth in size of the handling-marketing companies and their stable collaboration with farmers [18] is allowing them to develop organizational capacities to increase SASC volume: (1) strengthening of the organic range and expansion of their product portfolio; (2) optimization of order preparation, packaging, and packing for SASC; (3) improvement in digital marketing and consumer communication; and (4) upgrading customer service.

Currently, the development of the SASC hardly interferes with the traditional ASC, as more than 80% of its market is export, but the increase in volume and geographical extension of SASC may require its adaptation. SASC represents a very small volume of business in relation to the total turnover of the traditional ASC, although it does provide handling-marketing companies a global vision of the market, as it brings them closer to consumer. SASCs also have to be careful in their approach to the market so that large-scale distribution, which is the main customer of traditional ASC, does not perceive them as a threat.

SASCs results have yet to be evaluated in the medium and long term as they have many pending issues to improve: adjust its product portfolio, standardization of specific SASC processes (such as internal transport or order preparation), use of biodegradable or reusable packaging, or increase consumer loyalty. Many of these improvements will enable better planning and control of SASC.

According to [38], SFSCs have a positive effect in other territories outside Spain and Europe, such as Quebec. The author highlights that the most positive aspects of these chains are the adoption of sustainable agricultural practices, job creation and satisfaction, development of farmers’ skills, the economic weight of SFSCs within the local economy, the influence of SFSCs on access to fresh and healthy food, and their effects on social cohesion.

The study has focused on a fruit and vegetable cluster with specific characteristics [7]: short production cycle (allowing faster adaptation to the market), exhaustive quality control and traceability, great dynamism and a high level of technification and innovation, export orientation, or short life perishable products (which have to be brought quickly to the market). The specificity of the context of this study may make this model of SASC coexisting in a long SC (hybrid SC) not directly reproducible to other agricultural products or to other food supply chains such as meat, fish, oils and fats, and dairy products. Thus, ref. [4] in the case of meat and meat products, they note that intermediate sales of meat have increased in recent years. However, there is the problem of matching the slaughter of animals to consumer demand. The active use of social media, with its immediate dissemination of information, can enable producers to overcome this problem, creating an interactive SASC while offering consumers the opportunity to gain greater product knowledge.

## 5. Conclusions

The SASCs for fresh fruit and vegetables that are emerging in south-east Spain have their origin in handling-marketing companies which are farmers’ cooperatives or trading companies with close relationships with farmers. These handling-marketing companies, integrated in traditional ASC, have additionally developed their own SASC in contact with final consumer. SASC coexist with traditional ASC and share many stages of the SC leveraging convergence of interests developing hybrid SC. These hybrid SCs emerge from the supply side and can benefit from economies of agglomeration, scale, and scope that contribute to the sustainability of the agricultural model with various options to meet customer needs. For example, this type of hybrid SC facilitates the fight against food waste by quickly disposing of sizes or products that at a given moment do not meet the standards set in the long chain but are in perfect nutritional and physical condition.

Concentration in agricultural production allows effective and efficient access to the necessary advanced technologies (variety selection, biological pest control, plant nutrition, climate control, irrigation systems, fruit ripening control, etc.). Mass production for large, export-oriented SCs require homogeneity and compliance with specifications (dates, varieties, size, crop management, and traceability) which facilitates the reduction in production costs. However, agricultural production, because of its natural character, have components of heterogeneity (crop production is inherently subject to a multitude of changing environmental conditions). SASCs are more suited to variability in agricultural production, as their product specifications can be more flexible and change rapidly. The flexibility and agility of short chains allows access to customer segments that particularly value certain product attributes (freshness, taste, smell, size). According to [37], this intention to increase short supply chain businesses is an important trend in other countries such as Sweden. They even have policy instruments that encourage choices to increase consumer access and sales points (other than large retailers) to ensure that behaviors not only change along the supply chain, but also among consumers. They use systems such as food boxes and nearby farm shops. Harmonization between large and small-scale supply chain logistics and their use of infrastructure is necessary to implement greater energy efficiency and reach more consumers. Regional food-hubs have been successful in North America as an option to supply regional food on a larger scale without needing to change much of their logistics and infrastructure attractive to large-scale producers who lack niche markets on their farm or who simply wish to engage in short-term supply chain activities [37].

This new paradigm of hybrid chains in which short and long fruit SCs coexist is a sustainable innovation and it is beginning to set a clear trend in long fruit and vegetable SCs. Its results still need to be better assessed as some issues of SASC, such as the geographical scope or the range of products to be offered, are highly variable, despite the fact that in our study we found clear advantages derived from synergies in common processes.

## Figures and Tables

**Table 1:** Hybrid Agri-food Chains in Southeast Spain. Source: own production.

Agri-Food Supply Chain	X1	X2	X3	X4	X5	X6	X7	X8	X9
Matrix Chain Type	2nd grade cooperative	Cooperative	Public Limited Company S.A.	Agricultural Transformation Company S.A.T.	Cooperative	Cooperative	Limited Company S.L.	Cooperative	Public Limited Company S.A.
Type of Short Chain (SC)	Online Sale: box scheme	Online sales: box scheme	Online sales: box scheme	Online sale: box scheme	Online sales: box scheme	Collective farm shop	Online sales: box scheme	Fruit shop chain	Online sales
Year of implementation SC	2019	2018	2011	2018	2020	2019	2021	2011	2017
Range of products offered	Standard Fruit and Vegetable Boxes	Standard Fruit and Vegetable Boxes/Bulk Products	Standard Fruit and Vegetable Boxes/Boxes + Oil	Standard Fruit and Vegetable Boxes/Bulk products/V Range	Standard Fruit and Vegetable Boxes/ Bulk products V Range	Bulk products/V Range	Boxes of melons	Bulk products/V Range	V Range/Will include some fresh vegetables
Allows changes in delivery composition	No	Yes	No	Yes	Yes	Yes	Yes	Yes	Yes
Frequency of deliveries	1 day per week	2 days a week	1 day per week	Daily	Daily	Daily	Daily (Monday to Thursday)	Daily	Daily (Monday to Thursday)
Allows origin collection	No	Yes	Yes	Yes	Yes	Yes	Yes	Yes	Yes
Transport cost included	Yes	No	No	Yes (minimum order)	Yes (minimum order)	Not applicable	Yes	Not applicable	Yes
Price level	Competitive	Market price/Premium	Market price/Premium	Market price	Market price	Market price	Market price	Market price	Market price/Premium
Promotions	Yes	Yes	Not confirmed	Yes	Not confirmed	Not confirmed	Not confirmed	Not confirmed	Not confirmed
Quantity delivered	Fixed 5/6 kg	Variable	Between 3/7 kg	Variable	Variable	Variable	Between 5/12 kg	Variable	Variable
Delivery zone	Mainland Spain	Mainland Spain	Mainland Spain	Mainland Spain	Mainland Spain	Local	Mainland Spain	Local	Mainland Spain
Have online sales	Yes	Yes	Yes	Yes	Yes	No	Yes	No	Yes
Website or social networks	Yes	Yes	Yes	Yes	Yes	Yes	Yes	Yes	Yes
Possibility of subscription	Yes	No	No	No	No	No	No	No	No

**Table 2:** Proposed ASC Phases.

	Type of Expert					
Proposed	Supply Chain Professionals				Global Meeting	Final Proposal
Stage	Key Agents Study Case	Competitor Companies	Commercialize Association	University Teachers Expert		
1	Seed houses (including I + D and agricultural researchers and technicians)	Seed houses	Input: seeding	Input: Seeds., cultivation system, fertilizer, plant protection, water, climate, man power, machinery, energy sources.	Input: Seeds, biological control, Phytosanitary product, fertilizer, technologies, packaging companies.	Input: Raw materials, goods and services necessary for the cultivation and processing of fruit and vegetables.
2	Inputs acquisition	Farmer	Farmer: Plantation, cropping, harvesting	Primary Production	Primary production	Primary Production
3	Farmers	Producer Group	Farming cooperatives	Transport of raw material to the processing plant	Transport of raw material to the processing plant	Transport products to centers and Processing
4	Farming cooperatives (including the following services: agricultural technicians, quality assurance personnel, packing house managers, packing line managers, intake team, packers, dispatch personnel)	Packaging house	Packaging houses: handling	Processing	Processing	Order processing
5	Marketing team (including Marketing team, Business Development, Product Category Managers, Community Managers, Website programmers)	Marketing	Transport	Distribution	Marketing team and IT Services: Marketing team, business development, product category manager, community manager, website programmers,	Digital marketing and IT Services
6	Labeling Department	Business development	Direct sales	Selling point	Logistics	Distribution
7	IT Services	Sales	National sales	Consumer	Customers and Customer Service	Customers and Customer service
8	Logistics managers	Transport	International sales	--	--	--
9	Transport (GLS, DHL, MRW)	Customer	--	--	--	--
10	Customer Service and Dispute Team	After Sales	--	--	--	--

## Data Availability

Not applicable.
